# Multimodal pretraining for unsupervised protein representation learning

**DOI:** 10.1093/biomethods/bpae043

**Published:** 2024-06-18

**Authors:** Viet Thanh Duy Nguyen, Truong Son Hy

**Affiliations:** FPT Software AI Center, HCMC, Hanoi, Vietnam; FPT Software AI Center, HCMC, Hanoi, Vietnam; Department of Mathematics and Computer Science, Indiana State University, Terre Haute, IN, 47809, United States

**Keywords:** Protein representation learning, Unsupervised pretraining, Multimodal representation, Large Language Models, Geometric Deep Learning

## Abstract

Proteins are complex biomolecules essential for numerous biological processes, making them crucial targets for advancements in molecular biology, medical research, and drug design. Understanding their intricate, hierarchical structures, and functions is vital for progress in these fields. To capture this complexity, we introduce Multimodal Protein Representation Learning (MPRL), a novel framework for symmetry-preserving multimodal pretraining that learns unified, unsupervised protein representations by integrating primary and tertiary structures. MPRL employs Evolutionary Scale Modeling (ESM-2) for sequence analysis, Variational Graph Auto-Encoders (VGAE) for residue-level graphs, and PointNet Autoencoder (PAE) for 3D point clouds of atoms, each designed to capture the spatial and evolutionary intricacies of proteins while preserving critical symmetries. By leveraging Auto-Fusion to synthesize joint representations from these pretrained models, MPRL ensures robust and comprehensive protein representations. Our extensive evaluation demonstrates that MPRL significantly enhances performance in various tasks such as protein–ligand binding affinity prediction, protein fold classification, enzyme activity identification, and mutation stability prediction. This framework advances the understanding of protein dynamics and facilitates future research in the field. Our source code is publicly available at https://github.com/HySonLab/Protein_Pretrain.

## Introduction

Proteins, the essential building blocks of life, play a crucial role in a wide range of biological processes, rendering them a subject of profound scientific interest. Understanding the intricate structures and functions of proteins holds immense significance, yielding valuable contributions to numerous fields, such as molecular biology, medical research, and drug design [1, 2]. The advent of data-driven approaches, including machine learning (ML) and deep learning (DL), has revolutionized the field of protein research [3, 4]. These methods leverage data to unlock deeper insights into proteins, offering more precise predictions while significantly reducing the need for resource-intensive laboratory experiments.

While supervised representation learning in protein research has made considerable progress, its potential remains constrained by the limited availability of labeled data, a resource-intensive and time-consuming requirement. As a result, there is a growing interest in unsupervised pretraining methods, which can equip models with foundational knowledge of proteins without relying on extensive labeled datasets. Inspired by the remarkable success of unsupervised pretraining in domains like Natural Language Processing (NLP) [5] and generative AI [6], researchers have increasingly turned their attention to applying similar techniques to proteins, aiming to learn representations that capture both their structural intricacies and functional characteristics. These efforts have yielded notable achievements in advancing our understanding of proteins [7, 8].

**Figure 1.**
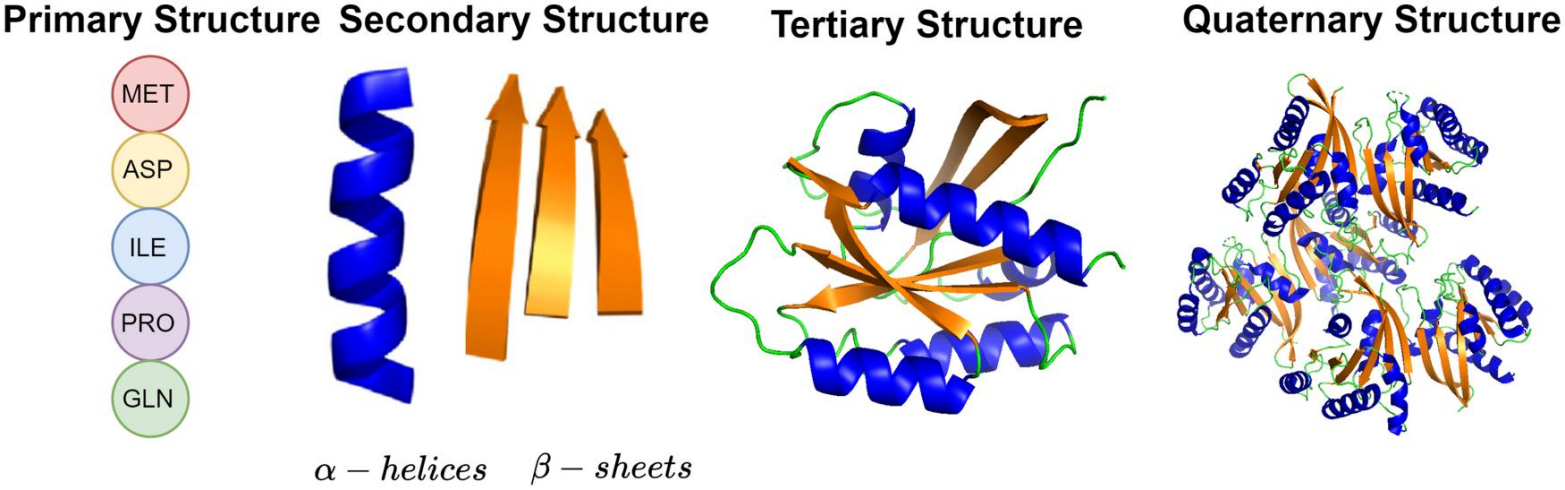
The four levels of protein structure are defined by the complexity within the polypeptide chain. Primary structure is determined by the specific sequence of amino acids linked together to form a protein. Secondary structure is characterized by the local folding and coiling of the polypeptide chain, which contributes to the protein’s 3D configuration. Tertiary structure encompasses the overall 3D shape of a single polypeptide chain. Quaternary structure is defined by the arrangement and interaction of multiple polypeptide chains within a larger protein complex.

Proteins are complex biomolecules with a hierarchical structure, consisting of four distinct levels: primary, secondary, tertiary, and quaternary (as illustrated in Fig. 1). Each of these structural levels corresponds to a specific modality of representation. While previous pretraining methods have typically treated these modalities in isolation, the complexity and multifaceted nature of proteins necessitates a more comprehensive and integrated approach to representation learning. Furthermore, these methods have commonly disregarded the critical aspect of preserving symmetries inherent to proteins, including rotation, and translation. Proteins exhibit symmetrical properties crucial to their biological functions, and failing to account for and maintain these symmetries can lead to inaccuracies in representation. To address the limitations of previous pretraining methods, we present Multimodal Protein Representation Learning (MPRL), an innovative framework designed for symmetry-preserving multimodal pretraining. This framework effectively learns unified, unsupervised representations of proteins by combining primary and tertiary structural data. MPRL utilizes Evolutionary Scale Modeling (ESM-2) [9] for analyzing sequences, Variational Graph Auto-Encoders (VGAE) [10] for processing residue-level graphs, and PointNet Autoencoder (PAE) [11] for managing 3D point clouds of atoms. Each method is tailored to grasp both the spatial and evolutionary complexities of proteins while maintaining essential symmetries. Utilizing Auto-Fusion [12], MPRL synthesizes a joint representation from these pretrained models, thereby facilitating effective intermodal information extraction and creating robust, comprehensive representations of proteins.

Our contributions can be summarized as follows:

- Introduced the MPRL framework, which utilizes unsupervised, symmetry-preserving pretraining methods tailored to each protein modality: ESM-2 for sequences, VGAE for residue-level graphs, and PAE for 3D point clouds, integrating these through Auto-Fusion to enhance intermodal information extraction.
- Conducted thorough evaluations of the MPRL framework, assessing its performance across pretraining tasks to ensure comprehensive validation.
- Validated the effectiveness of MPRL through extensive testing on several downstream tasks, including protein–ligand binding affinity prediction, protein fold classification, enzyme identification, and mutation stability prediction (MSP), demonstrating its broad applicability and robustness.

## Related work

### Unsupervised pretraining on proteins

Traditional supervised representation learning on proteins requires extensive labeled datasets, which are often costly and time-consuming to produce. While successful in specific applications [13–15], these methods generally yield representations that are overly specialized and lack generalizability across different protein-related tasks. In contrast, unsupervised pretraining methods offer a more scalable and versatile approach, enabling the learning of more generalized protein representations from abundant unlabeled data. Notably, previous research has effectively utilized unsupervised pretraining techniques tailored to specific modalities—such as sequences, graphs, and 3D point clouds—to enrich our understanding of proteins from different perspectives.

####  

##### Sequence-based pretraining

Learning from amino acid sequences is foundational in protein studies, as sequences encode the primary structure of proteins. Inspired by the success of NLP models, researchers have developed Protein Language Models (PLMs) such as ESM-1 [16], ESM-2 [9], ProtTrans [17], and ProteinBERT [18]. These models pretrain on vast datasets of sequences using tasks like masked-language modeling, allowing them to capture deep evolutionary and functional insights without relying on labeled data. While PLMs have achieved impressive results on a variety of downstream tasks for structure and function prediction [19, 20], they are bound by the inherent limitations of amino acid sequence data alone, which may not fully represent the complex spatial and dynamic properties of proteins.

##### Graph-based pretraining

Proteins can be effectively modeled as residue-level graphs, where nodes represent amino acids and edges reflect spatial or functional relationships. This method is particularly valuable because it captures the 3D structure of proteins, which is often obscured in their linear sequences. For example, residues that are distant in the sequence may be adjacent in the folded structure due to protein folding, where the sequence adopts a complex 3D shape. Research has explored the use of residue-level graphs for protein representation, with significant achievements in various protein-related tasks [21–23]. However, these models have limitations; although they excel at representing local interactions within protein structures, they often struggle to capture long-range interactions due to their focus on immediate connections. This can restrict their ability to model the full complexity of large protein structures, where distant amino acids may influence each other’s behavior [24, 25].

##### 3D point cloud-based pretraining

Although amino acid sequences and residue-level graphs are instrumental in understanding proteins, they fall short in depicting the detailed spatial arrangements and the complex folding patterns that define a protein’s 3D shape. In the field of computer vision, numerous unsupervised representation learning techniques that utilize 3D point cloud data have demonstrated significant advancements [26–28]. However, the adoption of these techniques in protein research remains limited. Our research bridges this gap by introducing an unsupervised pretraining method that leverages 3D point cloud data for proteins. This approach aims to harness the rich spatial information encoded in these data, thereby enhancing protein representation learning by providing a more precise visualization of protein structures and their dynamic changes.

### Symmetry-preserving models in protein representation

Symmetry in mathematics is a type of invariance, referring to the property that a mathematical object remains unchanged under a set of operations or transformations, which can be smooth, continuous, or discrete. Symmetries are crucial in many scientific problems and ML tasks due to their ability to maintain consistent properties despite changes in perspective or configuration. In graph representation learning, the scalar output of any graph neural network must be invariant with respect to the permutation of nodes [29, 30]. In chemistry and biochemistry, any neural network predicting the molecular properties must be rotationally invariant with respect to the molecule’s orientation in space [31, 32].

Despite these requirements, previous methods in protein representation have often neglected the critical aspect of preserving these inherent symmetries. This oversight can lead to models that, while potentially effective in narrow applications, fail to generalize across different tasks or conditions where symmetry plays a fundamental role. Our work addresses this gap by ensuring that our pretraining methods on residue-level graphs and 3D point clouds of the protein atoms respect both the rotation and translation symmetries. By preserving these critical symmetries, our models not only adhere more closely to the true nature of biological structures but also enhance their robustness and applicability across a broader range of protein-related tasks.

### Multimodal protein representation learning

Protein representation learning is inherently challenging due to proteins’ complex, hierarchical structures. While amino acid sequences provide primary structural insights, they often fail to capture complex spatial relationships. Residue-level graphs offer a better perspective on spatial proximity but may overlook long-range interactions. Conversely, 3D point clouds excel at depicting detailed spatial arrangements but lack the ability to directly capture sequence or residue-level details. To bridge these gaps, various multimodal learning approaches have been developed. Significant contributions in this area [15, 33, 34] have attempted to integrate different data modalities to enhance protein representation. However, these methods have not fully realized the potential of fully integrated modalities. Typically, they combine at most two modalities, which limits their ability to provide a comprehensive understanding of protein structures and functions. Our research advances this field by introducing a comprehensive framework that synergistically combines multiple modalities, aiming to improve the performance of various protein-related tasks.

## Method

**Figure 2.**
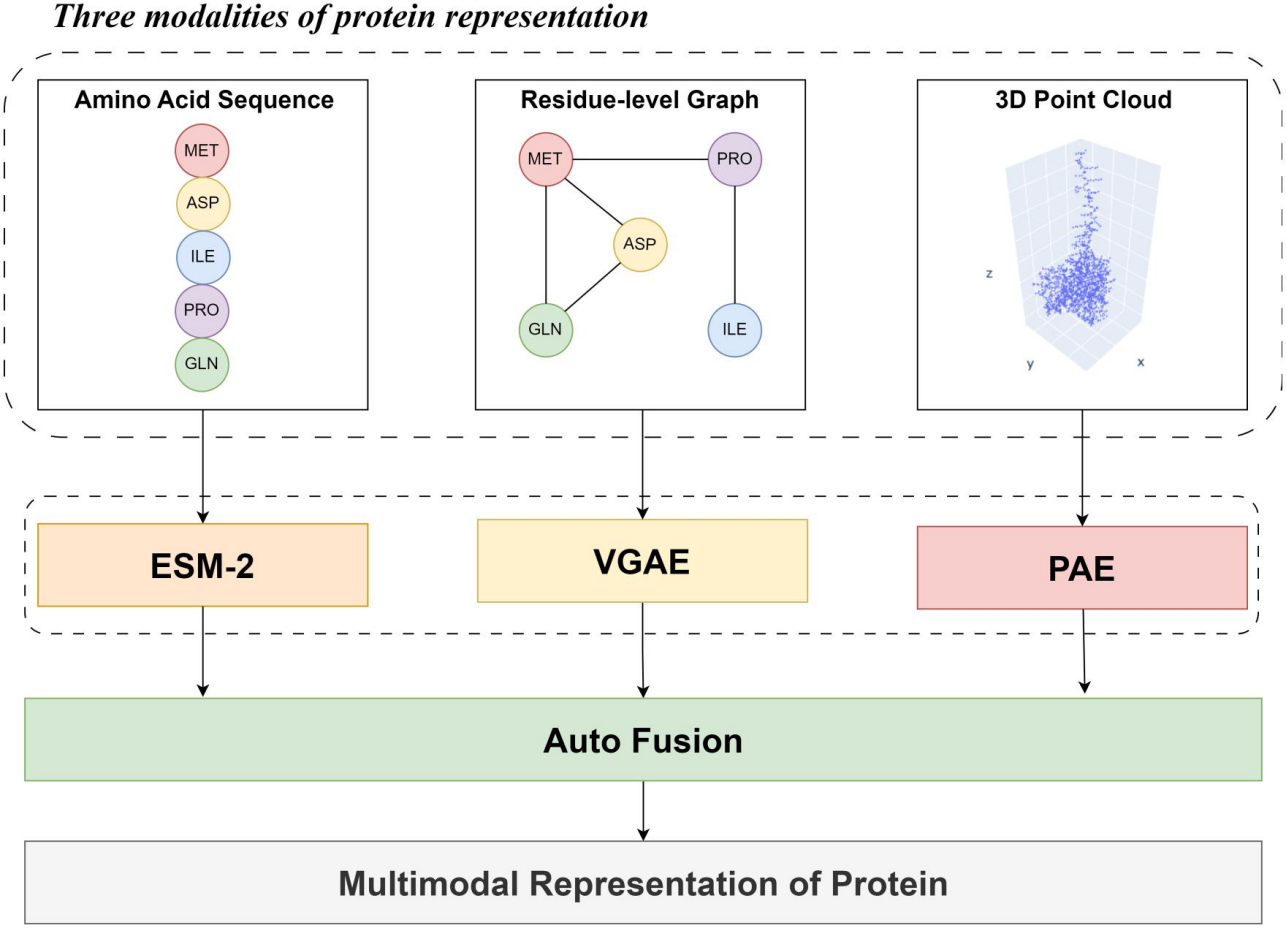
Overview of our MPRL framework.

Our framework (as illustrated in Fig. 2) leverages symmetry-preserving multimodal unsupervised pretraining on proteins to learn informative representations that capture the intricate structural and functional features of proteins. To this end, we develop specialized pretraining methods for each protein modality, ensuring that the learned representations preserve the symmetry of the protein. We employ ESM-2 to capture essential information from amino acid sequences. To comprehensively understand the intricate spatial relationships between amino acid residues, we apply VGAE to residue-level graphs. Additionally, we utilize PAE to extract spatial arrangements of atoms from 3D point clouds. Once we have obtained meaningful representations from these diverse pretraining strategies, the next critical step in our framework is to fuse these representations using Auto-Fusion. This fusion process aims to synthesize joint representations from the pretrained models, capturing and combining the essential aspects of protein structures from multiple modalities. The fused multimodal representation can then be leveraged for a variety of protein-related downstream tasks.

### Pretraining on sequences

#### ESM-2

To obtain protein sequence representations, we employ the well-established pretrained ESM-2 [9]. ESM-2, a recent development building upon the ESM-1 architecture, is a state-of-the-art transformer architecture that offers variations with parameter counts ranging from 8 million to a staggering 15 billion. It is trained on over 65 million unique protein sequences to predict the identity of randomly masked amino acids. By leveraging a massive-scale training approach that involves solving missing puzzles, ESM-2 is able to effectively internalize complex sequence patterns across evolution and generate high-quality embeddings that are rich in both evolutionary and functional insights. Notably, the process of generating ESM-2 embeddings for a protein sequence is significantly more efficient in terms of computational resources and time investment as it does not rely on multiple sequence alignments. ESM-2 embeddings are invaluable for various protein-related tasks, including structure prediction, design, and functional annotation, due to their computational efficiency and the profound evolutionary and functional insights they encapsulate.

#### Our ESM-2 embeddings process

In our approach, it is important to emphasize that we do not engage in training the ESM-2 model ourselves. Instead, we make efficient use of several pre-trained ESM-2 checkpoints. To maintain a balanced model complexity across our modalities, we opted for a medium-sized ESM-2 version, which comprises 150 million parameters. The process initiates with the tokenization of protein sequences, facilitated by the corresponding tokenizer. Subsequently, we feed these tokenized sequences into the ESM-2 model to generate encoded representations. Specifically, we extract the last hidden state from the final model layer to obtain the encoded sequences, encapsulating vital information about proteins. This approach not only conserves computational resources but also ensures a consistent level of model complexity across our modalities.

### Pretraining on residue-level graphs

#### Graph construction

To construct residue-level graphs, we employ a systematic approach to encapsulate the intricate spatial relationships between amino acid residues within protein structures. This process involves two key components:

##### Node representation

Within the residue-level graphs, each amino acid residue is treated as a node. These nodes are uniquely characterized through one-hot encoding, capturing the specific amino acid type for each residue. This representation provides a fundamental basis for the graph’s structural understanding.

##### Edge construction

Our residue-level graphs use a K-nearest neighbor (KNN) strategy to construct edges based on the spatial coordinates of the alpha carbon atoms of amino acid residues. This approach focuses on the central role of alpha carbons in the protein backbone, using Euclidean distance to connect each residue to its *k* nearest neighbors. The set of edges *E* is defined as:

$$E=\{(i,j)|d(i,j)\leq d_{k},i\neq j\}$$

where *d*(*i*, *j*) is the Euclidean distance between the alpha carbons of residues *i* and *j*, and *d_k_* is the distance threshold set by the *k*-th nearest neighbor. The distance *d*(*i*, *j*) is computed as:

$$d(i,j)=\sqrt{{\left(x_{i}-x_{j}\right)}^{2}+{\left(y_{i}-y_{j}\right)}^{2}+{\left(z_{i}-z_{j}\right)}^{2}}$$

with $\left(x_{i},y_{i},z_{i}\right)$ and $\left(x_{j},y_{j},z_{j}\right)$ representing the coordinates of the alpha carbons. We select *k *=* *5 to connect each node to its five nearest neighbors, balancing meaningful structural representation with computational efficiency. This approach to edge construction not only captures the immediate physical connections between amino acid residues but also reflects their functional relationships within the protein’s tertiary structure.

### VGAE

**Figure 3.**
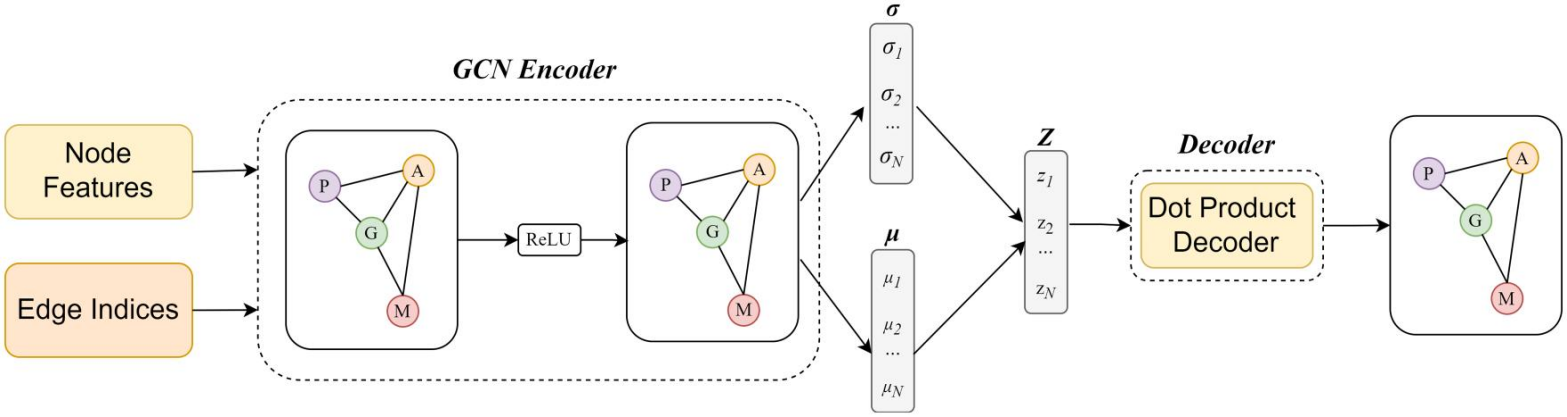
Architecture of the VGAE model. The input consists of edge indices and node features, composing a residue-level protein graph. The VGAE adopts a variational approach using the GCN Encoder, which is a key component of the model. In this encoder, GCN with two layers processes the input, applying ReLU activation, and produces both mean μ and log-variance log(σ) vectors. These vectors represent the parameters of the latent distribution. During training, latent vectors ***Z*** are sampled from this distribution, facilitating the learning of meaningful representations. The VGAE leverages this latent space to reconstruct the input graph through a dot product decoder.

In our pursuit of meaningful residue-level graph representations for protein representation learning, we utilize the VGAE model [10]. VGAE is a specialized framework for unsupervised learning on graph-structured data, building upon the principles of the variational autoencoder (VAE) [35]. This approach utilizes latent variables, empowering it to acquire interpretable latent representations tailored for undirected, unweighted graphs. Our VGAE architecture (as illustrated in Fig. 3) consists of a graph convolutional network (GCN) encoder and a straightforward inner product decoder. The GCN encoder learns to encode the residue-level graph into a latent representation that captures the important structural features of the protein. The inner product decoder then reconstructs the residue-level graph from the latent representation. We pretrain our VGAE model to learn meaningful latent embeddings on a link prediction task on a dataset of residue-level graphs constructed from a set of protein structures.

The primary objective during training is to minimize a reconstruction loss, which assesses the VGAE model’s ability to faithfully reconstruct the original residue-level graph from the learned latent representation. This loss computation involves the consideration of both positive edges, as specified by the provided edge index, and negative edges, which are randomly generated through a negative sampling process. The reconstruction loss can be mathematically expressed as follows:

$$L_{r}=-\sum_{i\in E^{+}}\;\text{log}\;(p(i|z))-\sum_{j\in E^{-}}\;\text{log}\;(1-p(j|z)),$$

where *E*^+^ is the set of positive edges in the original residue-level graph, *E*^–^ is the set of negative edges randomly generated through negative sampling, and p(i|z) is the probability of edge i being present in the reconstructed residue-level graph, given the latent representation z. While traditional VAE-based models incorporate the Kullback–Leibler divergence loss to encourage the latent distribution to align with a standard normal distribution, we intentionally exclude this component. This strategic choice aligns with our precise focus on protein structure representation, allowing the VGAE to serve this purpose effectively.

Notably, our VGAE model is designed to preserve essential protein symmetries, including rotation and translation, through specific architectural choices. First, the use of one-hot encoding for node features helps ensure that our model remains invariant to rotation and translation. One-hot encoding assigns a distinct value to each amino acid type, making the representation insensitive to the orientation and position of residues within the protein structure. Second, the KNN edge construction strategy, based on internode distances, supports invariance to both rotation and translation. This strategy effectively captures the spatial relationships between residues based on their relative distances, ensuring that edge connections remain consistent regardless of the protein’s orientation in space. Moreover, our model incorporates GCNs with a message-passing scheme [36] to encode the graphs at the residue level into a set of latent vectors. Each vector correlates with a specific residue, supporting permutational equivariance—an essential feature for processing graphs effectively. This property ensures that the output of our model remains consistent, regardless of the order in which nodes are processed.

### Pretraining on 3D point clouds

#### Point clouds construction

Point cloud construction is a process of converting protein structural data into a point cloud format, which accurately captures the 3D spatial distribution of atoms within protein molecules. Our systematic construction process involves the following key components:

##### Point representation

Within our constructed point clouds, each atom from the protein structure is individually represented as a point in 3D space, characterized by its specific coordinates (*x*, *y*, *z*). This detailed encoding captures the spatial identity of each atom and forms the fundamental basis for our point cloud representation, facilitating a comprehensive understanding of the structural intricacies within the protein.

Given a protein structure with *N* atoms, the point cloud can be represented as:

$$P=\{p_{i}|p_{i}=(x_{i},y_{i},z_{i}),i=1,2,\ldots ,N\}$$

##### Standardization

This process ensures uniform spatial distribution and size across all point clouds, which is crucial for consistent analysis across different protein structures.

- **Centering:** Each point cloud **P** is centered around the origin:
  $$c=\frac{1}{N}\sum_{i=1}^{N}p_{i},\;p_{i}^{\prime }=p_{i}-c$$
   This step translates the centroid of each point cloud to the origin.
- **Scaling:** The centered point cloud is scaled to fit within a unit sphere:
  $$r_{\text{max}}=\underset{i}{\max}||p_{i}^{\prime }||,\;p_{i}^{\prime \prime }\;=\frac{p_{i}^{\prime }}{r_{\text{max}}}$$
   This normalizes all point clouds to the same scale, ensuring no single structure dominates due to size.

##### Fixed number of points

Maintaining a consistent number of points in each point cloud is crucial for uniformity in model training.

- **Padding:** If *N* < *M*, the point cloud is padded with zero vectors until the total count reaches *M*.
- **Trimming:** If *N* > *M*, the point cloud is reduced to exactly *M* points.

By following these steps, we ensure that all point cloud representations have a consistent size and scale, facilitating reliable and comparable analyses across different protein structures.

### PAE

**Figure 4.**
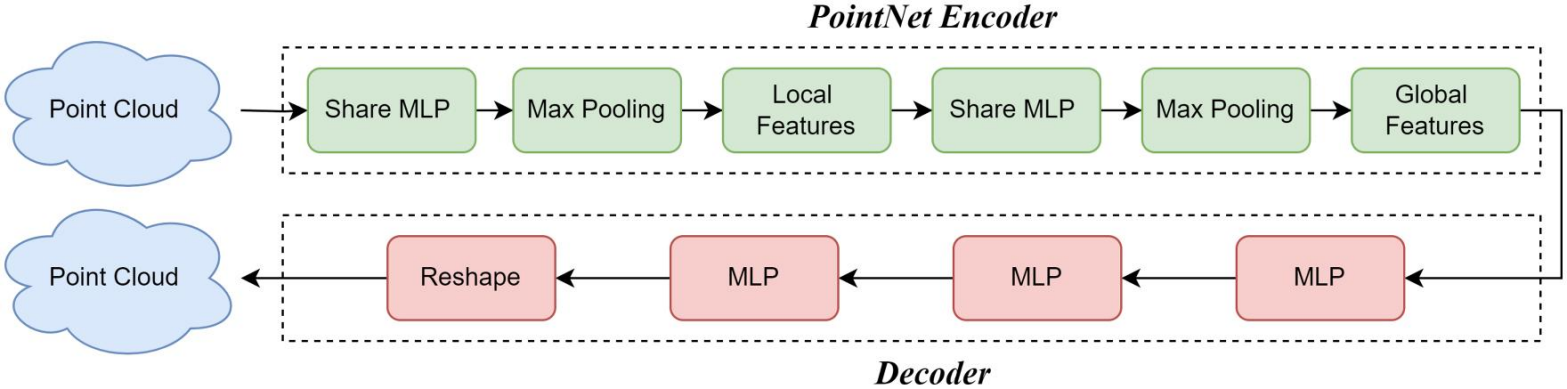
Architecture of the PAE model. The model consists of a PointNet-based encoder for capturing the structural information of the point cloud and a custom decoder for reconstructing the input point cloud from the encoded representation. The decoder is implemented as a multilayer perceptron (MLP) with hidden units of 512, 256, and 256 in its fully connected layers, each followed by ReLU activations and batch normalization. The final restoration is obtained by reshaping the output to match the specified number of points.

In our quest to derive informative 3D point cloud representations for protein representation learning, we adopt the PAE [11]. PAE, a specialized architecture designed for unsupervised learning using point cloud data, takes inspiration from the principles of the autoencoder while harnessing the robust capabilities of the PointNet framework [37]. Our PAE architecture (as illustrated in Fig. 4) consists of two fundamental components: the PointNet encoder and decoder. The encoder’s primary role is to capture and extract essential features from the input 3D point cloud data. Within this component, PointNet assumes a pivotal role, enabling the model to comprehend and represent intricate structural information. On the other hand, the decoder takes the encoded representations and efficiently reconstructs the 3D point cloud data. It employs several layers, including fully connected layers and batch normalization, to ensure the accurate restoration of the point cloud’s spatial information.

Our PAE was trained using the chamfer distance (CD) as the loss function. This metric is particularly effective for measuring the similarity between two point clouds and possesses the advantageous property of being invariant to the order of points within the clouds. The CD is calculated as the sum of squared distances between each point in one of the two point clouds and its nearest corresponding point in the other point cloud. This distance metric is expressed mathematically as follows:

$$\text{CD}(X,Y)=\sum_{x\in X}\underset{y\in Y}{\min}||x-y|{|}_{2}^{2}+\sum_{y\in Y}\underset{x\in X}{\min}||y-x|{|}_{2}^{2},$$

where *X* and *Y* are sets of points representing point clouds.

Our PAE model is specifically designed to maintain crucial protein symmetries, particularly rotation and translation, through carefully chosen architectural features. To address rotational symmetry, the model incorporates random rotations during training, enhancing its ability to recognize and adapt to various orientations of protein structures. For translational symmetry, a preprocessing step centers each input point cloud around the origin, focusing the model on relative spatial relationships rather than absolute positions. Moreover, the foundation of our PAE architecture is the PointNet framework, a permutation-equivariant neural network optimized for processing unordered point clouds. This design is made possible by the use of a symmetric max-pooling function, which ensures that the learned representations are invariant to the order of the points within the cloud—an essential feature for analyzing molecular structures where data points are not sequentially dependent.

### Multimodal fusion

#### Processing learned representations from pretrained models

A critical aspect of our approach is to harmonize the learned representations from various pretrained models to maintain balance across modalities. To achieve this, we ensure that all feature vectors obtained from different modalities have the same number of dimensionality. For the ESM and PAE models, their output is a feature vector consisting of 640 elements, but for the VGAE model, its output is a feature matrix with each row representing a feature vector for each node in the protein graph. To ensure uniform dimensionality, we employ the top-k pooling technique to select the top 640 nodes from the VGAE output matrix and compute an average feature vector. Furthermore, we standardize these feature vectors using z-score normalization to ensure that they are on the same scale and have the same distribution. This standardization improves the performance of the fusion model by preventing any single modality from dominating the fusion process.

#### Auto-fusion

**Figure 5.**
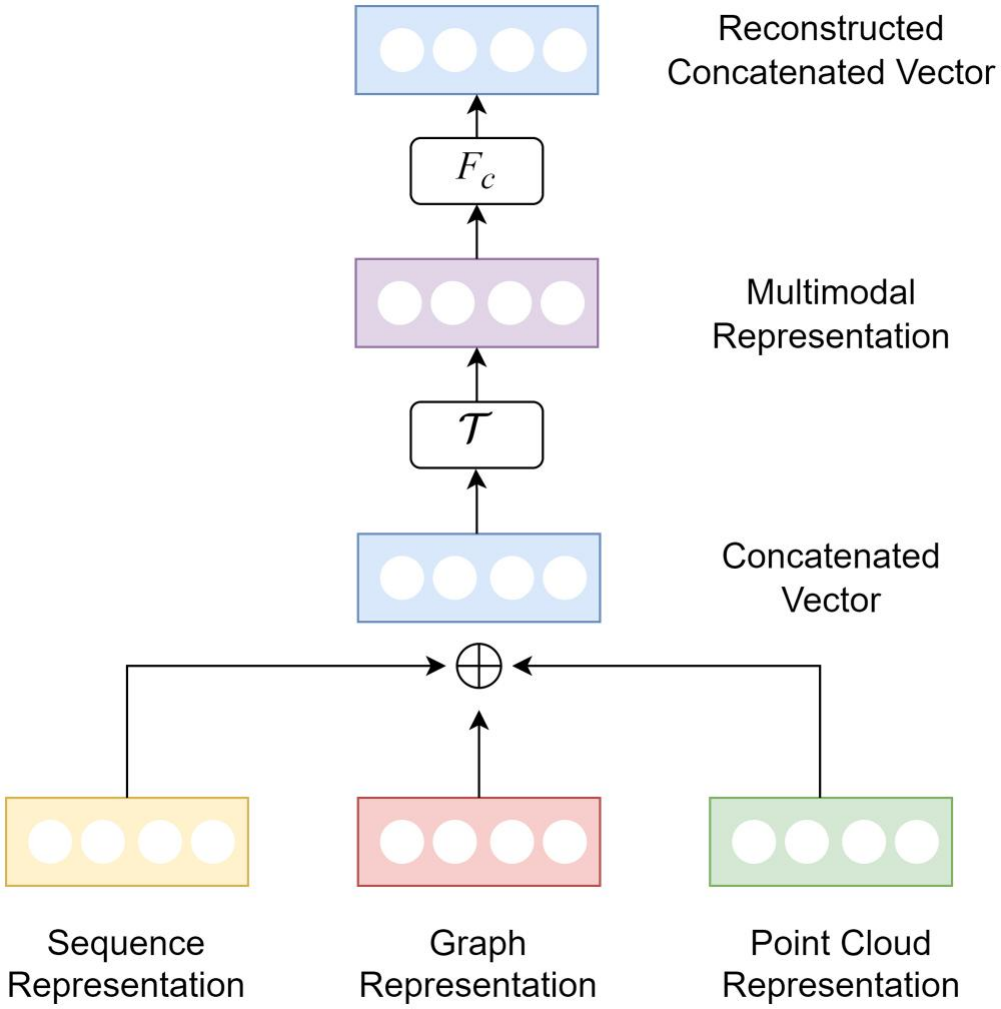
Architecture of the Auto-Fusion model. The sequence, graph, and point cloud representations are feature vectors generated by respective pretrained models for each modality. Initially, these vectors are concatenated and passed through a transformation ***T***, yielding a unified multimodal representation. The final reconstructed concatenated vector is obtained by processing this unified representation through another transformation, denoted as $F_{C}$.

In this study, we leverage Auto-Fusion [12] as our approach for multimodal synthesis, a method proven to enhance the model’s ability to extract intermodal features by optimizing the correlation between the different input modalities. Our Auto-Fusion architecture (as illustrated in Fig. 5) consists of two primary components: the input feature fusion module and the reconstruction module. The fusion process commences by concatenating individual unimodal feature vectors, each obtained from our distinct pretraining methods. These concatenated features undergo a series of transformations facilitated by linear layers and non-linear activation functions within the input feature fusion module, culminating in the generation of an autofused latent vector. Subsequently, the reconstruction module takes over. Its primary objective is to reconstruct the original concatenated feature vector from the autofused latent representation. This process entails reverse transformations that seek to minimize the Euclidean distance between the original and reconstructed concatenated vectors using the mean squared error (MSE), expressed as:

$$\text{MSE}(X,Y)=\frac{1}{n}\sum_{i=1}^{n}{\left(X_{i}-Y_{i}\right)}^{2}.$$

Notably, this reconstruction loss function plays a crucial role in refining the fused representation. Auto-Fusion ensures that the learned autofused vector retains only the essential shared information from the input modalities. It achieves this by effectively eliminating any arbitrary signals that might originate from the individual unimodal features. This meticulous optimization process enhances the quality of the fused representation, making it highly effective for synthesizing multimodal data. Our choice of Auto-Fusion aligns seamlessly with our goal of achieving a balanced and informative fusion of pretraining strategies in our framework.

## Experiments

In this section, we thoroughly evaluate the performance of our MPRL framework. Our evaluation begins with a detailed pretraining analysis, leveraging a comprehensive dataset of protein structures to ensure the model’s robustness in capturing complex structural features. Subsequently, we assess the pretrained models on four pivotal downstream tasks: protein–ligand binding affinity prediction, protein fold classification, enzyme identification, and MSP. Each task employs a diverse and widely recognized dataset, allowing for a rigorous examination of our approach’s generalizability and effectiveness. Additionally, we present further experiments validating the symmetry-preserving capabilities of our model in Appendix 3 and assessments using a leak-free dataset in Appendix 4. Detailed implementation specifics, including hyperparameters and training processes, are provided in Appendix 1, while a comprehensive description of the evaluation metrics utilized in our study can be found in Appendix 2.

### Pretraining evaluation

#### Material

To facilitate robust unsupervised training, a substantial dataset comprising a multitude of PDB files is essential to ensure our model captures the intricate structure of proteins. In this endeavor, we utilized the Swiss-Prot structure dataset, which was sourced directly from the AlphaFold Protein Structure database. This dataset, generated by the AlphaFold platform, one of the state-of-the-art methods for protein folding, contains 542 378 PDB files, representing a diverse range of proteins from a variety of organisms. This makes it an ideal dataset for unsupervised pretraining, as it allows the model to learn a generalizable representation of protein structures. To organize this vast dataset effectively, we randomly divided it into subsets, comprising a training set, a validation set, and a test set. This division follows a balanced ratio of 70:20:10, enabling us to train, validate, and evaluate our model on distinct portions of the dataset and ensure its robustness and generalization. For each of the subsequent pretraining steps, including VGAE, PAE, and Auto-Fusion, we maintained a consistent pretraining duration of 100 epochs. This standardized approach ensures that all models had an equal opportunity to learn informative protein representations during the pretraining phase.

#### Pretraining evaluation on VGAE

During the training process, both the training loss and validation loss consistently decreased, indicating effective learning. The final training loss and validation loss for VGAE converged to 0.951 and 0.952, respectively. For performance evaluation, VGAE was assessed on the link prediction task using the test dataset. The results indicate a remarkable achievement, with an area under the curve of 0.95 and an average precision of 0.97. These metrics emphasize VGAE’s effectiveness in capturing essential structural features in protein graphs and demonstrate its ability to predict protein interactions accurately.

#### Pretraining evaluation on PAE

Throughout the training process, the training loss exhibited a consistent and significant decrease, demonstrating the model’s ability to effectively comprehend intricate protein structures. However, the validation loss curve displayed some instability during this phase, albeit with an overall trend toward decreasing values compared to the initial levels. The final training loss and validation loss for PAE were 6.51 and 7.84, respectively. The relatively high variance observed in the validation loss curve could potentially be attributed to several factors, such as the complexity and diversity within the protein structure dataset. The PAE model achieved a CD of 7.89 on the test set for the task of reconstructing the 3D point cloud of proteins. This is an outstanding result that demonstrates the ability of the PAE model to learn informative representations of protein structures, even for proteins with complex and diverse structures.

#### Pretraining evaluation on auto-fusion

During the training process of Auto-Fusion, the training and validation loss consistently decreased over time, albeit with occasional fluctuations. The final training loss and validation loss for Auto-Fusion converged to 0.03 and 0.299, respectively. This behavior indicates the model’s adaptability to complex protein structures while maintaining a general decreasing trend in loss values. Auto-Fusion’s performance was further assessed using a dedicated test dataset, with a focus on its capability to reconstruct the original concatenated feature vector from the auto-fused latent representation. Remarkably, Auto-Fusion achieved an exceptional MSE of 0.03. This low MSE value emphasizes the model’s efficacy in preserving essential structural features during the reconstruction process, solidifying Auto-Fusion’s role as a robust component in our framework for protein representation learning.

### Fine-tuning results

#### Protein–ligand binding affinity prediction

##### Problem statement

Structure-based drug design, a powerful approach for identifying potential drug candidates, involves assessing the fit and interactions of small molecules (ligands) within the binding sites of target proteins [38]. The strength of these interactions, known as binding affinity, is a key determinant of a ligand’s ability to modulate the protein’s biological function [39]. Therefore, compounds with high binding affinity to target proteins are prioritized as potential drug candidates. The accurate prediction of binding affinity is essential for efficiently screening compound libraries and optimizing lead compounds, thereby reducing the costs of drug discovery.

##### Material

To gage the effectiveness of our multimodal representation in predicting protein–ligand binding affinity, we conducted assessments using three distinct ligand-binding datasets: DAVIS [40], KIBA [41], and PDBbind version 2020 [42]. The DAVIS dataset encompasses 442 proteins and 68 ligands, forming a dataset comprising 30 056 protein–ligand binding pairs. In DAVIS, the binding scores are quantified as KD constants. Conversely, the KIBA dataset is characterized by 229 proteins and an expanded collection of 2111 ligands, creating a dataset that consists of 118 254 protein–ligand binding pairs, with binding affinities represented as KIBA scores. The PDBbind version 2020 dataset extends our evaluation with a rich repository of 19 433 protein–ligand binding pairs, divided into a general set with 14 127 samples and a refined set with 5316 samples. This dataset provides experimentally measured binding affinities for protein–ligand interactions, encompassing data on peptides and nucleic acids expressed in units like Kd, Ki, or IC50, or their negative logarithmic equivalents (Pkd). The incorporation of these diverse datasets forms a comprehensive and varied tested, allowing us to thoroughly assess the predictive capabilities of our multimodal representation when estimating protein–ligand binding affinities. To ensure a fair and standardized evaluation, we meticulously followed the test/training/validation split settings as outlined in previous studies, specifically adhering to the configurations defined in the respective sources for the DAVIS, KIBA, and PDBbind version 2020 datasets [15, 43]. By maintaining this consistency, we aimed to create a level playing field for comparisons, allowing for an equitable assessment of our multimodal representation’s performance. Furthermore, we adopted the same measurement metrics used in these previous works, aligning our methodology with established standards to facilitate straightforward and meaningful comparisons with existing research outcomes. This rigorous approach enhances the reliability and comprehensibility of our results within the broader scientific community.

##### Method

For each data point within these datasets, we used a hybrid approach that combined the protein representation from our framework with the ligand representation from Morgan fingerprints. We concatenated these two distinct representations into a single feature vector, which was then used as input to our Gaussian Process (GP) Regressor model. The GP Regressor is a powerful ML model that leverages the principles of GPs for regression tasks. It is particularly well-suited for modeling complex relationships between input features and target values. By employing GPR as our modeling technique, we harnessed its ability to capture intricate patterns within the data, enabling us to make accurate predictions while quantifying the associated uncertainty. This approach allowed us to effectively estimate protein–ligand binding affinities and assess ligand–protein interactions within the given datasets.

#### Protein fold classification

##### Problem statement

Protein fold classification plays a crucial role in unraveling the profound interplay between protein structure, function, and the evolutionary trajectory of biological molecules. It enables the grouping of proteins into specific fold classes, drawing upon common attributes such as their secondary structure composition, spatial arrangements, and connectivity patterns. This classification process is indispensable for the comprehensive understanding of proteins. By categorizing them into fold classes, we gain insights into their functional characteristics, allowing us to decipher the intricate relationship between form and function. Additionally, it provides valuable information about how proteins have evolved over time. In light of these considerations, our primary objective in this context is to accurately predict the fold class to which a given protein belongs.

##### Material

For the protein fold classification task, we utilized the SCOPe version 1.75 dataset, as established in Hou *et al*. [44], which offers well-defined training, validation, and test partitions. This dataset encompasses a comprehensive collection of 16 712 proteins originating from 1195 unique protein folds. The 3D structural information for these proteins was sourced from the SCOPe 1.75 database, as provided by [45]. The dataset comprises three distinct test subsets: “Fold” where proteins from the same superfamily are excluded from the training set; “Superfamily” in which proteins from the same family are omitted from the training data; and “Family” wherein proteins from the same family are retained within the training set.

##### Method

For each data point within this dataset, we exclusively leveraged the multimodal representation of proteins generated by our framework. This representation served as the sole input for our XGBoost Classifier model. XGBoost Classifier is a powerful ML algorithm that is based on the gradient boosting principle. Gradient boosting is an ensemble learning technique that combines the predictions of multiple weak learners to produce a more accurate prediction. By utilizing the XGBoost Classifier as our chosen model, we harnessed its strength in making accurate and robust predictions, even when dealing with intricate protein structural data. This approach allowed us to effectively classify proteins into their respective fold classes based on the provided multimodal representations. To assess the performance of our model in protein structural classification, we employed the accuracy metric.

#### Enzyme identification

##### Problem statement

There are seven primary protein categories that encompass all proteins, each serving a unique function in biological processes. These categories consist of antibodies, contractile proteins, enzymes, hormonal proteins, structural proteins, storage proteins, and transport proteins. Among these diverse protein types, enzymes hold a pivotal position. Enzymes are proteins whose function is to catalyze, or accelerate, specific chemical reactions within the cell. The accurate identification of enzymes within the larger spectrum of proteins is fundamental to understanding the intricate biochemical workings of life and has significant implications for various fields, such as biotechnology and medicine.

##### Material

In this task, we utilized the D&D benchmark dataset defined by Dobson and Doig [46], which consists of 1178 structurally diverse proteins, comprising 691 enzymes and 487 non-enzymes, based on annotations in the PDB and Medline abstracts. To ensure a fair and consistent comparison with prior research, we adopted the 10-fold cross-validation partitioning established in Hermosilla *et al*. [47].

##### Method

Similar to our approach to protein fold classification, we utilized our multimodal representation learning framework to extract informative protein representations for the enzyme identification task. These representations were then fed into an XGBoost Classifier model to predict whether a given protein is an enzyme or not. Our approach allowed us to effectively classify proteins into their respective categories, distinguishing between enzymes and non-enzymes based on the provided multimodal representations. To measure the performance of our model in this task, we calculated the average accuracy across the 10-fold cross-validation setup.

#### MSP

##### Problem statement

Predicting the stability of protein mutations is a critical task in understanding the intricate interplay between genetic variations and protein structure and function. Mutations can profoundly alter protein stability, leading to changes in their structural dynamics and, consequently, affecting their biological activities. Accurately predicting the stability of mutated proteins is essential for designing novel proteins. While experimental techniques for probing these changes are labor-intensive, the development of efficient computational methods presents a promising alternative. To address this challenge, the task is formulated as a binary classification problem, aiming to predict whether the stability of the protein complex increases or decreases as a result of the mutation.

##### Material

The dataset for the MSP task is sourced from Atom3D [48], a comprehensive benchmark encompassing both novel and established datasets spanning several critical classes of biomolecules. Each mutation in the MSP task includes a PDB file with the residue of interest transformed to the specified mutant amino acid, as well as the native PDB file. A total of 4148 mutant structures accompanied by their 316 wild-type (WT) structures are provided. For labeling, a value of 1 is assigned to a mutant if the dissociation constant (Kd) of the mutant protein is less than that of the WT protein, indicating improved binding; otherwise, a label of 0 is assigned. To ensure that the model is not simply memorizing the training data, protein complexes are such that no protein in the test dataset has more than 30% sequence identity with any protein in the training dataset.

##### Method

To tackle the MSP task, we employed our multimodal representation learning framework to extract informative representations of both WT and mutant proteins. These representations encapsulate the structural and functional characteristics of the proteins, providing the necessary information to predict the stability changes induced by mutations. Subsequently, the extracted multimodal representations were concatenated to form a single, combined representation. This concatenation process enabled the model to integrate information from both WT and mutant proteins, allowing it to learn the relationships between their representations and predict mutation stability outcomes. The concatenated multimodal representation was then fed into an XGBoost Classifier model. The choice of the area under the ROC curve (AUROC) as the evaluation metric stems from its suitability for imbalanced datasets, ensuring robust performance assessment in the context of the inherent data imbalance.

### Discussion

Our experiments demonstrate the effectiveness of our MPRL framework in generating informative protein representations. This framework, which uses unsupervised pretraining models, consistently delivers impressive results across four critical downstream tasks: protein–ligand binding affinity prediction (see Tables 1 and 2), protein fold classification (see Table 3), enzyme identification (see Table 4), and MSP (see Table 5). Specifically, MPRL excels in the MSP task, achieving the highest AUROC of 0.612, slightly surpassing the top baseline’s 0.609. In protein–ligand binding affinity, although our model does not surpass the leading models, it remains competitive, trailing them by narrow margins of MSE 0.248 compared to 0.202 in DAVIS, MSE 0.199 compared to 0.155 in KIBA, and root mean square error (RMSE) 1.372 compared to 1.314 in PDBBind version 2020. In enzyme identification, MPRL achieves an accuracy of 83.9%, just behind the best-performing model at 85.5%. Similarly, in protein fold classification across three test subsets—Fold, Superfamily, and Family—MPRL demonstrates its robustness with scores of 43.1%, 63.2%, and 98.4%, respectively, closely trailing the top-performing models which achieve 45.0%, 69.7%, and 99.1%. These results underscore MPRL’s capability to adapt effectively across a diverse array of protein-related tasks, nearly matching the performance of the top specialized models. This adaptability highlights the framework’s broad applicability without the need for specific tuning for individual tasks, providing a balance between generalizability and specialization. Another key aspect of our approach is the use of traditional ML methods, such as GP and XGBoost, to leverage our multimodal protein representations. Despite the complexity of the tasks, these conventional ML models deliver highly favorable results, showcasing the generality and informativeness of our protein representations. Additionally, these methods enhance model interpretability, which is crucial in scientific research where understanding the underlying factors driving predictions is vital.

**Table 1.** Experimental results of protein–ligand binding affinity prediction task on DAVIS and KIBA dataset.

Approach	DAVIS			KIBA		
	MSE↓	CI↑	$r_{m}^{2}↑$	MSE↓	CI↑	$r_{m}^{2}↑$
KronRLS [49]	0.379	0.871	0.407	0.411	0.782	0.342
SimBoost [50]	0.282	0.872	0.640	0.220	0.836	0.629
SmCNN-DTA [51]	0.319	0.852	0.590	0.274	0.821	0.573
DeepDTA [52]	0.261	0.878	0.630	0.194	0.863	0.673
WideDTA [53]	0.886	0.202	–	0.875	0.179	–
AttentionDTA [54]	0.216	0.893	0.670	0.155	0.882	0.755
MATT-DTI [55]	0.227	0.891	0.653	**0.150**	0.882	0.756
GraphDTA [56]	0.258	0.884	0.656	0.162	0.879	0.736
FusionDTA [57]	0.220	0.903	0.666	0.167	**0.891**	0.699
BiCompDTA [58]	0.237	0.904	0.696	0.167	**0.891**	0.757
PMN [15]	**0.202**	**0.906**	**0.739**	0.153	0.874	**0.767**
ESM-2	0.300	0.690	0.311	0.258	0.789	0.326
VGAE (ours)	0.508	0.631	0.032	0.344	0.752	0.250
PAE (ours)	0.558	0.636	0.042	0.329	0.749	0.252
MPRL (ours)	0.248	0.699	0.414	0.199	0.812	0.471

We highlight the best result for each metric in bold.

**Table 2.** Experimental results of protein–ligand binding affinity prediction task on PDBBind version 2020 dataset.

Approach	RMSE↓	MAE↓	Pearson↑	Spearman↑	$r_{m}^{2}↑$	CI↑
Pafnucy [59]	1.435	1.144	0.635	0.587	0.348	0.707
OnionNet [60]	1.403	1.103	0.648	0.602	0.381	0.717
IGN [61]	1.404	1.116	0.662	0.638	0.385	0.73
SIGN [62]	1.373	1.086	0.685	0.656	0.398	0.736
SMINA [63]	1.466	1.161	0.665	0.663	0.391	0.74
GNINA [64]	1.740	1.413	0.495	0.494	0.209	0.674
dMaSIF [65]	1.450	1.136	0.629	0.588	0.347	0.71
TankBind [66]	1.345	1.060	**0.718**	**0.689**	0.404	0.750
GraphDTA [67]	1.564	1.223	0.612	0.570	0.306	0.703
TransCPI [68]	1.493	1.201	0.604	0.551	0.255	0.677
MolTrans [69]	1.599	1.271	0.539	0.474	0.242	0.666
DrugBAN [70]	1.480	1.159	0.657	0.612	0.319	0.72
DGraphDTA [71]	1.493	1.201	0.604	0.551	0.312	0.693
WGNN-DTA [70]	1.501	1.196	0.605	0.562	0.311	0.697
STAMP-DPI [72]	1.503	1.176	0.653	0.601	0.327	0.719
PSICHIC [43]	**1.314**	**1.015**	0.710	0.686	**0.428**	**0.751**
ESM-2	1.380	1.120	0.659	0.619	0.247	0.719
VGAE (ours)	1.485	1.209	0.594	0.547	0.246	0.690
PAE (ours)	1.466	1.158	0.604	0.557	0.248	0.696
MPRL (ours)	1.372	1.104	0.663	0.634	0.246	0.726

We highlight the best result for each metric in bold.

**Table 3.** Experimental results of Protein fold classification task on SCOPe 1.75 dataset, reported in terms of Accuracy

Approach	Fold (%)	Superfamily (%)	Family (%)
GCNN [73]	25.7	46.5	95.9
ExConv [74]	30.1	46.3	92.0
InConvC [47]	37.6	65.1	98.7
InConvH [47]	40.8	62.0	98.4
InConvCH [47]	43.5	66.7	98.7
Ours3DCH [47]	40.7	62.2	98.1
CovNeigh [47]	27.2	41.5	92.3
HyNeigh [47]	33.3	50.6	96.9
NoPool [47]	37.1	59.8	97.6
GridPool [75]	28.6	41.8	91.8
TopKPool [76]	40.7	65.4	98.4
EdgePool [77]	44.4	69.6	99.0
RosettaCEN [78]	41.7	66.5	98.9
AminoGraph [47]	39.6	64.7	**99.1**
AtomicGraph [47]	**45.0**	**69.7**	98.9
ESM-2	40.2	61.2	98.4
VGAE (ours)	36.4	57.9	96.8
PAE (ours)	39.5	60.0	97.9
MPRL (ours)	43.1	63.2	98.4

Results are presented for three distinct test subsets: Fold, Superfamily, and Family.

We highlight the best result for each metric in bold.

**Table 4.** Experimental results of Enzyme identification task on D&D dataset.

Approach	Accuracy↑ (%)
Gao and Ji [79]	84.4
Ying *et al*. [80]	82.1
Zhao and Wang [81]	82.0
Nguyen *et al*. [82]	81.2
Zhang *et al*. [83]	79.7
Togninalli *et al*. [84]	79.4
Hermosilla *et al*. [47]	**85.5**
ESM-2	82.7
VGAE (ours)	81.4
PAE (ours)	80.4
MPRL (ours)	83.9

We highlight the best result in bold.

**Table 5.** Experimental results of MSP task on Atom3D.

Approach	AUROC↑
3DCNN [48]	0.574
GNN [48]	0.609
ENN [48]	0.574
Rao *et al*. [85]	0.554
ESM-2	0.509
VGAE (ours)	0.463
PAE (ours)	0.488
MPRL (ours)	**0.612**

We highlight the best result in bold

As evidenced by our ablation study, ESM-2 is the best-performing model among our pretraining models for each protein modality, maintaining a high level of performance across all tasks. This is largely due to its ability to leverage pre-trained contextual embeddings, which encapsulate the rich and intricate language of proteins. Currently, ESM-2 is considered one of the best approaches for obtaining protein representations, as it effectively captures evolutionary patterns and biochemical properties from large-scale protein sequence data. The strong performance of the sequence-only model in tasks that rely heavily on structural information, such as protein–ligand binding affinity prediction and protein fold classification, can be attributed to the rich contextual embeddings generated by ESM-2. Despite lacking direct structural data, ESM-2’s embeddings capture implicit structural cues embedded within the sequence data. The model learns patterns and relationships within protein sequences that correlate with 3D structural features, enabling ESM-2 to make accurate predictions for tasks traditionally dependent on explicit structural information. However, despite the strong performance of ESM-2, our MPRL model consistently demonstrates even better results, proving the effectiveness of the multimodal approach. The integration of multiple modalities allows our MPRL model to capture a more comprehensive range of protein features, leading to more robust and accurate predictions across various tasks. Although VGAE and PAE may not achieve outstanding results individually, they contribute unique information that ESM-2 may not capture. VGAE captures graph-based relationships between residues, while PAE focuses on spatial configurations through point clouds. These additional insights help the MPRL model to surpass ESM-2, highlighting the advantage of combining different types of information to achieve superior performance in protein representation learning.

## Conclusion

In this article, we have introduced MPRL, a comprehensive framework for protein representation learning, addressing critical challenges in the field. Our contributions encompass the development of dedicated unsupervised symmetry-preserving pretraining methods for distinct protein modalities, utilizing ESM-2 for amino acid sequences, VGAE for residue-level graphs, and PAE for 3D point clouds. Leveraging Auto-Fusion, our approach synthesizes joint representations, facilitating effective intermodal information extraction. Our experimental results demonstrate the effectiveness of multimodal unsupervised symmetry-preserving pre-training methods for learning protein representations, which is evidenced by our framework’s impressive performance on several protein-related downstream tasks, including protein–ligand binding affinity prediction, protein fold classification, enzyme identification, and MSP. Our framework’s ability to offer informative protein representations presents exciting opportunities for researchers to tackle complex problems in the realm of protein science.

## Data Availability

The data underlying this article are available in the article and in its online supplementary material.

## Appendix 1: Implementation details

This section provides an overview of the implementation details, with a focus on the key hyperparameters used in the models and the training process for our study. Understanding these details is essential for replicating and comprehending our experiments.

### Pretraining process

For the pretraining phase, we leveraged the ESM pretrained model, specifically opting for the 150 million parameter version available through Hugging Face’s Transformers [86] library. Our implementation of the VGAE, PAE, and Auto-Fusion models leveraged the PyTorch [87] and PyTorch Geometric [88] DL frameworks. Each pretrained model was configured to encode proteins into feature vectors, each comprising 640 elements. Subsequently, these individual feature vectors generated by the three models were synthesized into a unified feature vector with 1024 elements by our Auto-Fusion model. In terms of model training parameters, we conducted training over 100 epochs with a batch size of 256, employing a fixed learning rate of 0.001 and the Adam optimization algorithm. To enhance the training process’s effectiveness, we ensured random shuffling of the training data before initiating the training process.

### Downstream tasks

For our downstream tasks, we employed two well-established ML algorithms: GP and XGBoost. The GP model found its application in protein–ligand binding affinity prediction, while the XGBoost model was utilized for both protein fold classification and enzyme identification. The GP model was implemented using the GPyTorch [89] library, a robust tool for constructing and training GP models within the PyTorch framework. The GP model employed the Gaussian likelihood, a fitting choice for regression tasks, and the Radial Basis Function (RBF) kernel. The selection of the RBF kernel was motivated by its ability to capture smooth and continuous variations in data, aligning well with the typical characteristics of protein–ligand binding affinities. The training process for the GP model aimed at optimizing model parameters to effectively fit the training data. To achieve this, a negative log-likelihood loss function was employed, and the GP model was trained for 100 epochs, utilizing a batch size of 256 and the Adam optimization algorithm. This iterative training process enabled the model to learn underlying data patterns and make accurate predictions for unseen protein–ligand binding affinities. In parallel, we configured the XGBoost [90] model with specific hyperparameters, including a learning rate of 0.1, a maximum tree depth of 3, and a total of 1000 estimators. During the training process, we integrated evaluation metrics, with multi-class logarithmic loss serving as the chosen evaluation metric to monitor the model’s performance. To prevent overfitting, early stopping was implemented, with a tolerance of 10 rounds. This configuration ensured the XGBoost model’s effectiveness in the tasks of classifying protein folds and identifying enzymes.

## Appendix 2: Evaluation metrics

In this study, we utilize several key evaluation metrics to assess the performance of our models. Below, we explain each metric in detail:

### MSE

MSE measures the average of the squares of the errors between predicted and actual values, providing a sense of the variance in the errors. It is defined by the formula:

$$\text{MSE}=\frac{1}{n}\sum_{i=1}^{n}{\left(y_{i}-{\hat{y}}_{i}\right)}^{2}$$

where *n* is the number of samples, *y_i_* is the observed value, and ${\hat{y}}_{i}$ is the predicted value.

### RMSE

RMSE is the square root of the MSE, offering a measure of the standard deviation of the prediction errors. It is defined as:

$$\text{RMSE}=\sqrt{\frac{1}{n}\sum_{i=1}^{n}{\left(y_{i}-{\hat{y}}_{i}\right)}^{2}}$$

### MAE

MAE calculates the average magnitude of the errors in a set of predictions, without considering their direction. It is defined by:

$$\text{MAE}=\frac{1}{n}\sum_{i=1}^{n}|{\hat{y}}_{i}-y_{i}|$$

### Pearson correlation coefficient

Pearson’s correlation coefficient measures the linear relationship between two datasets, providing a value between −1 and +1, where 1 indicates a perfect positive linear correlation, −1 is a perfect negative linear correlation, and 0 is no linear correlation. It is defined as:

$$r=\frac{\sum \left(x_{i}-\bar{x}\right)(y_{i}-\bar{y})}{\sqrt{\sum {\left(x_{i}-\bar{x}\right)}^{2}\sum {\left(y_{i}-\bar{y}\right)}^{2}}}$$

where *x_i_* and *y_i_* are individual sample points, and $\bar{x}$ and $\bar{y}$ are the means of the *x* and *y* values, respectively.

### Spearman’s rank correlation coefficient

Spearman’s rank correlation assesses the strength and direction of the association between two ranked variables. It is defined as:

$$\rho =1-\frac{6\sum d_{i}^{2}}{n(n^{2}-1)}$$

where *d_i_* is the difference between the ranks of corresponding variables, and *n* is the number of observations.

### Concordance index (CI)

CI evaluates the predictive accuracy of a risk score model, indicating the probability that, in a randomly selected pair, the individual with the higher predicted risk experiences the event first. It is given by:

$$\text{CI}=\frac{1}{Z}\sum_{\delta_{i}>\delta_{j}}h({\hat{y}}_{i}-{\hat{y}}_{j})$$

where ${\hat{y}}_{i}$ denotes the prediction for the larger affinity *δ_i_*, ${\hat{y}}_{j}$ is the predicted value for the smaller affinity *δ_j_*, and *Z* is the normalization constant. The function *h*(*x*) is defined as:

$$h(x)=\{\begin{array}{cc} 1 & \text{if}\;x>0\\ 0.5 & \text{if}\;x=0\\ 0 & \text{if}\;x<0 \end{array}$$

### Adjusted *R*^2^ (*r*^2^_*m*_)

Adjusted *R*^2^ adjusts the *R*^2^ value for the number of predictors in a model, providing a more accurate measure of the proportion of variance explained by the independent variables. It is calculated as:

$$r_{m}^{2}=r^{2}\times \left(1-\sqrt{r^{2}-r_{0}^{2}}\right)$$

where *r*^2^ and $r_{0}^{2}$ are the squared correlation coefficients with and without intercepts, respectively.

### Accuracy

Accuracy measures the proportion of true results (both true positives and true negatives) among the total number of cases examined. It is defined as:

$$\text{Accuracy}=\frac{\text{Number}\;\text{of Correct Predictions}}{\text{Total Number of Predictions}}$$

### AUROC

AUROC evaluates the ability of a model to distinguish between classes, plotting the true positive rate (TPR) against the false positive rate (FPR) at various threshold settings. It is given by:

$$\text{AUROC}=\int_{0}^{1}\text{TPR}(t)d\text{FPR}(t)$$

where TPR(t) is the TPR and FPR(t) is the FPR at threshold *t*.

These metrics collectively provide a comprehensive evaluation of our model’s performance across various aspects, including error magnitude, correlation, and classification effectiveness.

## Appendix 3: Ablation experiments on symmetry preservation

This section details the ablation experiments conducted to empirically validate the symmetry-preserving capabilities of our model, as highlighted in the main sections of the article. The primary objective of these experiments is to assess whether our model can effectively retain symmetry information and maintain consistent performance across downstream tasks, particularly when faced with datasets that have undergone random rotations and translations. These transformations simulate real-world variations in data presentation, thereby testing the model’s robustness and reliability in practical applications. For this demonstration, we focus exclusively on protein–ligand binding affinity prediction due to its high sensitivity to the spatial configuration of protein and ligand molecules, making it an ideal test for evaluating the model’s ability to preserve symmetry information under transformation.

### Data preparation

To simulate real-world variations, each protein structure was randomly rotated and translated using the transformation:

$$p^{\prime }=Rp+t$$

where **p** is the original coordinates of the atoms in the protein, *R* is the rotation matrix, and **t** is the translation vector.

### Rotation matrix R

The rotation matrix *R* is constructed from Euler angles:

$$R=R_{z}(\psi )R_{y}(\phi )R_{x}(\theta )$$

where *R_x_*(*θ*)$,R_{y}(\phi ),$ and $R_{z}(\psi )$ are rotation matrices around the x, y, and z axes, respectively. The angles *θ*, ϕ, and *ψ* are chosen uniformly at random from the interval [0,2π].

### Translation vector t

The translation vector **t** applies a linear shift to the protein coordinates and is defined as:

$$t=(t_{x},t_{y},t_{z})$$

where *t_x_*, *t_y_*, and *t_z_* are components randomly selected from a uniform distribution within the range [−a,a], ensuring each protein is translated within a controlled spatial range.

### Results and discussion

The results presented in Table A1 illustrate the robustness of our model in handling transformations such as random rotations and translations. For the DAVIS and KIBA datasets, the RMSE values on the transformed datasets are very close to those on the original datasets, with increases of just 0.006 and 0.005, respectively. This indicates that the model’s performance is minimally affected by the spatial transformations, maintaining high accuracy across different orientations and positions of the protein–ligand complexes. Similarly, the PDBBind dataset shows a slight increase in RMSE from 1.372 to 1.380. While this represents the largest increase among the three datasets, it is still a marginal change, suggesting that the model preserves its predictive capabilities even under more complex and varied structural transformations inherent in larger datasets like PDBBind. These findings underscore the model’s effectiveness in utilizing symmetry information to maintain accuracy across spatial variations. This capability is crucial for real-world applications, where experimental data often come with inherent positional and orientational variabilities.

## Appendix 4: Experiment on leak-free dataset

This section describes additional experiments conducted using the LeakProof PDBBind dataset [91]. The primary purpose of these experiments is to validate our model’s performance using a dataset-split methodology specifically designed to eliminate potential data leaks. Such leaks can occur due to structural similarities between training and test set complexes, which might inadvertently lead to overestimated performance metrics and reduced model generalizability. Employing a leak-free dataset helps ensure that our model’s efficacy and robustness are genuinely reflective of its ability to generalize to unseen data.

The results presented in Table A2 underscore the effectiveness of the MPRL model in handling the protein–ligand binding affinity prediction task, particularly when evaluated using a rigorously curated leak-free dataset. Notably, our MPRL model achieves the lowest RMSE scores across all dataset splits (train, validation, and test), indicating a high level of accuracy and robustness. This performance is especially significant when compared to traditional and widely used models such as AutoDock Vina, IGN, RF-Score, and DeepDTA.

**Table A1.** RMSE comparison for protein–ligand binding affinity prediction on original and transformed datasets.

Dataset	Original	Transformed
DAVIS	0.497	0.503
KIBA	0.446	0.451
PDBBind version 2020	1.372	1.380

The RMSE values reflect the model’s performance accuracy in predicting binding affinities under both standard and altered spatial conditions.

**Table A2.** Experimental results of protein–ligand binding affinity prediction task on Leak Proof PDBBindt, reported in terms of RMSE.

Model	Train	Validation	Test
AutoDock Vina [92]	2.42	2.29	2.56
InteractionGraphNet (IGN) [56]	1.65	2.00	2.16
Random Forest (RF)-Score [93]	0.68	2.14	2.10
DeepDTA [52]	1.41	2.07	2.29
MPRL (Ours)	**0.48**	**1.47**	**1.55**

We highlight the best result in each data split in bold.
